# A spatial network analysis of resource partitioning between bumblebees foraging on artificial flowers in a flight cage

**DOI:** 10.1186/s40462-019-0150-z

**Published:** 2019-02-21

**Authors:** Cristian Pasquaretta, Raphael Jeanson, Jerome Pansanel, Nigel E. Raine, Lars Chittka, Mathieu Lihoreau

**Affiliations:** 1 Research Center on Animal Cognition (CRCA), Center for Integrative Biology (CBI); CNRS, University Paul Sabatier, Toulouse, France; 2 Institut Pluridisciplinaire Hubert Curien, CNRS, Strasbourg, France; 3 School of Biological Sciences, Royal Holloway University of London, Egham, TW20 0EX UK; 4 School of Environmental Sciences, University of Guelph, Guelph, Ontario N1G 2W1 Canada; 5 Department of Biological and Experimental Psychology, School of Biological and Chemical Sciences, Queen Mary University of London, Mile End Road, London, E1 4NS UK; 6 Wissenschaftskolleg, Institute for Advanced Study, Wallotstrasse 19, 14193 Berlin, Germany

**Keywords:** Bipartite networks, Bumble bees, Modularity, Plant-pollinator interactions, Resource partitioning, Social interactions

## Abstract

**Background:**

Individual bees exhibit complex movement patterns to efficiently exploit small areas within larger plant populations. How such individual spatial behaviours scale up to the collective level, when several foragers visit a common area, has remained challenging to investigate, both because of the low resolution of field movement data and the limited power of the statistical descriptors to analyse them. To tackle these issues we video recorded all flower visits (*N* = 6205), and every interaction on flowers (*N* = 628), involving foragers from a bumblebee (*Bombus terrestris*) colony in a large outdoor flight cage (880 m^2^), containing ten artificial flowers, collected on five consecutive days, and analysed bee movements using networks statistics.

**Results:**

Bee-flower visitation networks were significantly more modular than expected by chance, indicating that foragers minimized overlaps in their patterns of flower visits. Resource partitioning emerged from differences in foraging experience among bees, and from outcomes of their interactions on flowers. Less experienced foragers showed lower activity and were more faithful to some flowers, whereas more experienced foragers explored the flower array more extensively. Furthermore, bees avoided returning to flowers from which they had recently been displaced by a nestmate, suggesting that bees integrate memories of past interactions into their foraging decisions.

**Conclusion:**

Our observations, under high levels of competition in a flight cage, suggest that the continuous turnover of foragers observed in colonies can led to efficient resource partitioning among bees in natural conditions.

**Electronic supplementary material:**

The online version of this article (10.1186/s40462-019-0150-z) contains supplementary material, which is available to authorized users.

## Background

Foraging theory predicts that animals should self-distribute on food resources in order to maximise their individual energy intake rates [1, 2]. Accordingly, many animals have evolved strategies to assess the quality of food resources and avoid competition, either in the form of exploitative competition (when the activity of other foragers reduces the availability of resources) or interference competition (when physical interactions between individuals affect access to resources) [3]. While most studies have focused on these competitive effects in situations when foragers decide to exploit one of many feeding sites [4], in nature, animals often face the challenge of exploiting multiple feeding sites simultaneously.

Social pollinators, such as bees, can individually visit hundreds of feeding locations (flowers, inflorescences, or flower patches) spread across large spatial scales, whose nectar rewards renew over time [5, 6]. Most detailed studies of bee spatial foraging patterns have been conducted in simplified experimental conditions using artificial flowers [7–9]. Individual bumblebees and honey bees with exclusive access to a stable array of flowers for which nectar rewards are regularly renewed, often learn the shortest possible route to visit as many flowers needed once and return to their nest [7, 10, 11]. Under most natural conditions, however, bees may face considerable additional variation in floral rewards due to the activity of other foragers exploiting the same resources, and this may importantly influence their spatial decisions. In principle, animals foraging on multiple resources that each provide a limited amount of food are expected to reduce the degree of spatial overlap among themselves to maximize their individual foraging efficiency [1, 2].

Previous studies on resource use by multiple bees indicate that individuals tend to adjust the size of their foraging area in response to changes in resource quality and the density of foragers [12–17]; either by increasing the number of plants (or flowers) they visit when competing foragers are removed and new areas become available (i.e. competitive vacuums) [18, 19], or by reducing the number of plants they visit after new foragers are introduced [20]. So far, all these observations have been conducted on focal individuals within larger populations of foragers [15, 18, 19] or on pairs of bees [12, 20], raising the question of how individual responses to competition influence foraging patterns at the population level.

**Fig. 1:**
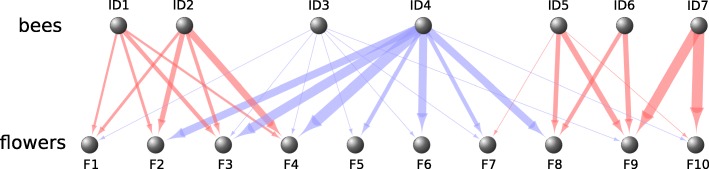
Schematic representation of a bee-flower bipartite network. Bees are active nodes (ID1-ID7) and flowers are passive nodes (F1-F10). Arrows represent the frequency of flower visits by each bee. In this hypothetical example, pink arrows indicate more flower-specific bees that tend to visit only a few flowers. Blue arrows indicate less flower-specific bees that visit nearly all available flowers

Network theory has recently emerged as a promising tool to identify and compare the statistical properties of pollinator movements at both the individual and collective level [21, 22]. Bee movements can be depicted as a two-mode (bipartite) network, in which several bees (active nodes) connect plants or flowers (passive nodes: see example in Fig. 1). The “modularity” of such a network describes the tendency of bees to exploit distinct groups of flowers (modules) [23, 24]. Modularity thus provides a measure of the level of resource partitioning among bees, and how this might change over time across successive networks. Analyses of field data suggest that the foraging patterns of bumblebees (and some other pollinators) are significantly modular and that the properties of plants (including species, numbers of flower heads, plant height) influence the emergence of plant-pollinator modules [15, 25]. However, current field data provide incomplete information about the density of foragers, the foraging experience of each individual bee, the location of their nest relative to different plants, their interactions on plants and the temporal dynamics of their foraging patterns. All these parameters are important for understanding how exploitative and interference competition may favour (or limit) resource partitioning among bees.

Here, we address this issue by developing an experimental and analytical approach to studying the dynamics of resource use by multiple bumblebees foraging in a controlled, yet ecologically relevant, experimental environment consisting of ten artificial flowers regularly distributed within a large outdoor flight cage. The number of flowers, their spatial distribution and nectar refill rates were chosen to promote potential competition among bees. We monitored all flower visits and interactions on flowers made by foragers from a bumblebee colony during five consecutive days. We analysed individual movement patterns and interactions on flowers using bee-flower bipartite networks to characterize the levels of resource partitioning among bees and how these changed over time.

## Methods

### Bees and flight cage

We conducted the study in September 2010 in a large flight cage (length = 44 m, width = 20 m, height = 3 m, mesh size = 0.5 mm; see Additional file 1: Figure S1) erected on a flat pasture at the Centre for Agricultural Bioscience International (CABI) in Egham (Surrey, UK). We obtained bees from a four-week old, commercially reared, *Bombus terrestris* colony (Syngenta Bioline Bees, Weert, The Netherlands) containing a queen, brood and about 200 workers. We marked all workers with individually numbered tags (*Opalith Plättchen,* Christian Graze KG, Germany) on their thorax within a day of emergence from pupae. We connected the colony nest box to a colourless transparent Plexiglas entrance tube and provided the colony with *ad libitum* defrosted honey bee-collected pollen directly into the nest box. Foragers collected sucrose solution (40% *v*/v) from artificial flowers in the flight cage.

### Artificial flowers

Each flower consisted of a landing platform, an electric syringe pump, a webcam and their supports (Additional file 1: Figure S2). The landing platform was a blue plastic disc (diameter = 60 mm) mounted horizontally on top of a clear plastic cylinder (height = 75 mm). A yellow circle (diameter = 20 mm) in the centre of the blue disc highlighted the location of the feeding cup (capacity = 40 μL) from which bees could collect sucrose solution. The feeding cup was connected to an electric syringe pump via a transparent plastic tube (internal diameter = 1 mm, length = 200 mm). As the pump depressed the syringe plunger, sucrose solution was pushed through the tube and accumulated in the feeding cup at a rate of 3.3 μL/min. The landing platform and the plastic cylinder were placed on a clear plastic support (length = 300 mm, width = 200 mm, height = 180 mm) directly on the ground.

### Video tracking

We mounted a motion sensitive video camera (webcam) on each artificial flower to automatically record bee visits [9, 12]. The webcam (Logitech c250, Fremont, CA) was fitted with a neutral density filter (Neutral Density = 0.6, Lee Filters, Andover, UK) to reduce the amount of light entering the lens. Each webcam was powered by a laptop computer running motion detection software (Zone Trigger 2, Omega Unfold, Quebec, Canada) programmed to record a video clip (minimum duration 5 s) every time a bee moved into the camera field of view until movement stopped. We therefore captured complete flower visits from the moment a bee landed to its departure. Viewing the landing platform from above enabled us to identify bees (from their dorsal numbered tags), their arrival and departure times, and whether they collected sucrose solution from the feeding cup. Video clips in which two bees visited the flower simultaneously provided data about the nature and frequency of behavioural interactions between foragers on flowers (18% of the 5180 clips). Video clips with three or more bees on the same flower were rare (1.1% of the 5180 clips) and difficult to interpret as all bees would often interact simultaneously. We therefore removed these videos from the analyses. We also mounted a webcam above the nest box entrance tube to record all arrivals at, and departures from, the colony by bees.

### Experimental procedure

**Fig. 2:**
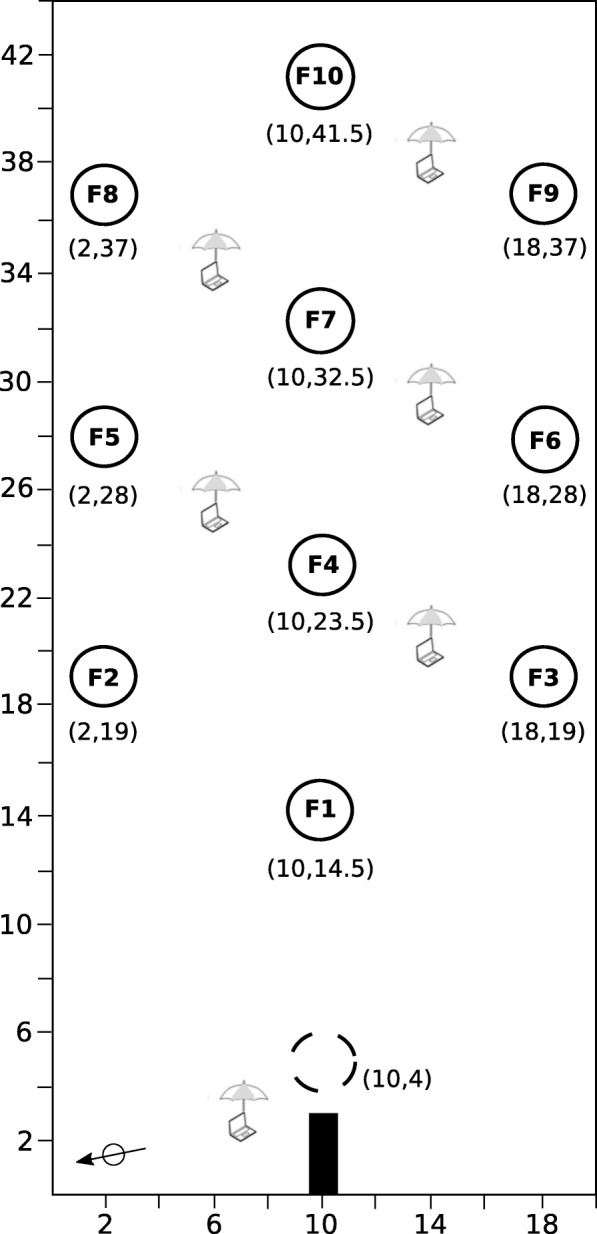
Spatial arrangement of the artificial flowers in the flight cage. Coordinates (x,y) of the colony nest box (black rectangle), the pre-training flower (dashed line circle) and the test flowers (plain circles F1-F10) are in metres. The distance between the nest box and F1 was 12.5 m. The distance between nearest neighbour flowers was 9 m (e.g. F1 and F4). The black arrow (bottom left) indicates North. Laptop computers, used to power the webcams on flowers and at the nest entrance, were protected from sun and rain by golf umbrellas. Photos of the flight cage and artificial flowers are shown in Additional file 1: Figures S1 and S2

We sat up a regular polygonal array of 10 flowers in the flight cage (Fig. 2 and Additional file 1: Figure S1, see also [12]). Nearest neighbour flowers were 9 m apart (e.g. F1 and F4) and second nearest neighbour flowers were 15.8 m apart (e.g. F1 and F6). Previous work indicates that because bumblebees are unable to detect reflecting (non self-luminant) visual targets presented against a vegetation background subtending a visual angle of ca. 3° [26], the maximum distance at which a forager could distinguish an artificial flower (overall dimension: length = 400 mm, width = 300 mm, height = 500 mm) from the background is assumed to be 9.6 m. Therefore, it is likely that bees visiting a flower could only detect nearest neighbour flowers. We used six laptop computers to operate the 11 webcams and record video data. Laptops were protected from sun and rain by golf umbrellas (height = 1.5 m, diameter = 1.0 m) that could be used by the bees as three-dimensional landmarks (Fig. 2 and Additional file 1: Figure S2).

We conducted the study during a period of six consecutive sunny days (with a clear blue sky) to minimize effects of weather variation. On day 1, we opened the colony and allowed all bees to forage freely on a pre-training flower placed 1 m in front of the nest box entrance (Fig. 2). This flower had the same shape and colour as the test flowers, but differed slightly as the feeding cup was filled with a cotton wick dipped into a reservoir of sucrose solution underneath the landing platform from which bees could extract *ad libitum *food until they were satiated. During this pre-training phase, test flowers, laptops and umbrellas were covered with black cloth bags so that all bees remained naive to test conditions. On days 2–6, we opened the colony entrance with all test flowers, laptops and umbrellas uncovered, and let bees forage *ad libitum* for six consecutive hours per day (10:30–16:30 GMT). Each morning, we started the syringe pumps 5 min before releasing the bees (opening the colony entrance) to provide a full reward of 20 μL of nectar in the feeding cup of each flower at the start of data collection. The number of flowers in the array and their high reward refill rate was chosen in order to generate appreciable levels of exploitative and interference competition among bees. In these conditions, bees made an average of 3.68 ± 0.95 (mean ± SD) unique flower visits per foraging bout. At the end of day 6, all bees were frozen (− 20 °C) and measured (thorax width).

### Data analyses

#### Flower visitation matrices

We ran all analyses in R (version 3.2.3). We extracted all flower visits made by each bee over the five days of the experiment from the video data. Because we were particularly interested in studying the movement patterns of foragers, we only retained the data from motivated bees that made more than 20 flower visits in total (i.e. 10 out of the 40 bees that left the colony at least once during the five days; see Additional file 2: dataset S1). To account for inter-individual variability in the foraging activity of bees we divided the overall flower visitation dataset into 20 sub-datasets representative of 20 different time intervals. Each of these time intervals was defined by the time taken by at least one bee to complete eight foraging bouts (in a foraging bout a bee leaves the nest, visits a series of flowers to fill its crop, then returns to the colony nest box), which varied from 50 to 116 min (*N* = 881 foraging bouts). This criterion was chosen based on the observation that bumblebees required a minimum of eight foraging bouts to develop stable flower visitation sequences in an array of five distant flowers [10]. Therefore, each bee could have completed between zero and eight foraging bouts during a given time interval. From these sub-datasets, we built 20 flower visitation matrices with flower identity in rows and bee identity in columns.

#### Activity, experience and flower specificity

For each of the 20 flower visitation matrices, we measured the level of activity, experience and flower specificity of each individual forager. “Activity” was the sum of all flower visits within each bin of eight foraging bouts performed by an individual bee: the more flowers a bee visited, the higher its activity. “Experience” was the cumulative activity of each bee during all its previous bins of eight foraging bouts (all flower visits since the start of the experiment). “Flower specificity” was the coefficient of variation of the frequency distribution of flower visits [27] calculated for each bin. This coefficient was normalized [28] so that specificity ranged between 0 (low flower specificity: a bee visits all 10 flowers) and 1 (high flower specificity: a bee visits only one flower). We verified whether flower specificity was affected by individual experience and activity by applying a linear mixed effect model (LMM), using the function *lmer* in the R package ‘lme4’ [29], including individual identity as random effect. Because activity and experience are correlated, we calculated the variance inflation factor (VIF) to remove one of the two variables in case the VIF value was found to be higher than 2 [30].

#### Network modularity

For each of the 20 flower visitation matrices, we constructed a bee-flower bipartite network in which bees are active nodes and flowers are passive nodes (see example in Fig. 1). We calculated the modularity of these bipartite networks to estimate the level of resource partitioning among bees and how this changes through time. Modularity measures the degree to which a network is organized into modules (sub-networks) of highly inter-connected nodes. Nodes of the same module are more connected to each other than nodes of different modules. Here, modularity measures the degree to which bees (nodes of the networks) use a specific set of flowers (interactions among nodes), and at which frequency, accounting for the total number of visits performed (i.e. the marginal total for rows and columns of the bee-flower matrix). Modularity ranges from 0 (no modules) if all bees visit all flowers at similar rates, to 1 (no connection between modules) if each bee uses a separate subset of flowers.

We calculated the average modularity for each of the twenty networks obtained, using the DIRTLPAwb+ algorithm [31], which accounts for the frequency of flower use by each bee. Because modularity depends on the size of the network (number of bees foraging at the same time) and the number of links (number of flowers visited) [32], we compared the modularity of each experimental network with the modularity of simulated networks obtained from 100 random matrices built by reshuffling the flower visitation matrices while keeping column and row marginals constant [33]. We compared the observed and simulated modularities using their standardized z-scores [34]. We ran a regression model (LM) to evaluate the effect of mean individual activity and experience on network modularities calculated for each bin of eight foraging bouts, including the number of individuals foraging as a covariate. We also tested for co-linearity among predictors using the VIF method described above.

#### Persistence of bee-flower associations

To quantify variation in bee-flower associations through time, we identified bee-flower modules for each network and counted the number of times each bee was associated with each flower in their respective module across all twenty networks. We then compared this measure between networks to investigate changes through time. We estimated whether there were significant differences between expected and observed frequencies using chi-squared tests for count data.

#### Interactions on flowers

**Fig. 3:**
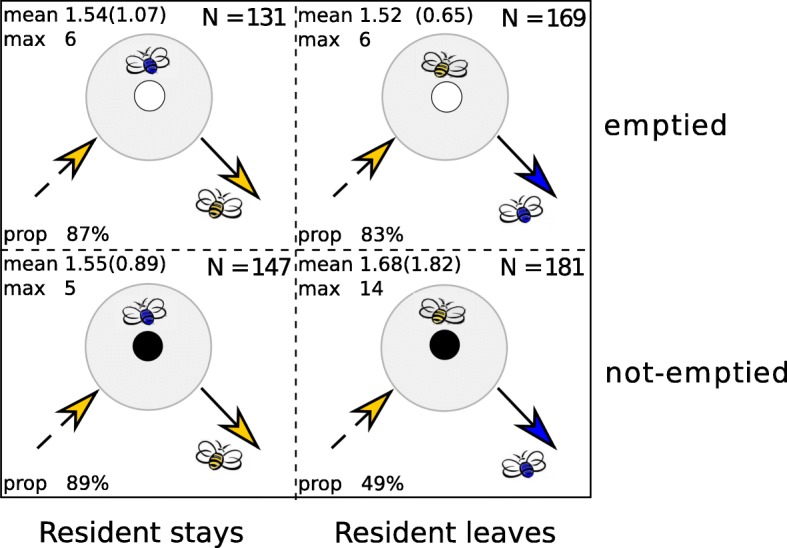
Social interactions on flowers. During an interaction a (yellow) bee joins another (blue) bee already on the flower (grey circle). The joining bee can leave the flower first (resident stays) or replace the resident bee (resident leaves). Both types of interactions can take place on emptied flowers (the resident bee fed on the flower for more than 8 s before the interaction occurred; grey circle with white centre) or on not-emptied (or partially emptied) flowers (the resident bee fed on the flower for less than 8 s before the interaction occurred; grey circle with black centre). For each type of interaction, the sample size, the mean duration (± standard deviation), the maximum duration, and the proportion of interactions where contacts occurred are shown

We conducted all interaction analyses on the same ten bees as the network analyses (see above). From the video clips, we identified two types of interactions on flowers (Fig. 3): “resident stays” (a bee landed on an already occupied flower and left first, either spontaneously or after being pushed; see example Additional file 3: Video S1); “resident leaves” (a bee landed on an already occupied flower and left second, effectively replacing the other bee that left either spontaneously or after being pushed; see example Additional file 4: Video S2). When physical contact occurred between two bees on the same flower, we calculated both the time the resident bee spent on the flower before the arrival of the second bee and the duration of the interaction between bees (i.e. the period when both bees were simultaneously on the flower).

For each interaction, we also estimated the reward status of the flower based on the amount of sucrose solution it contained when the interaction occurred. Since a lone bee on a flower consumed 40 μL of sucrose solution (full feeding cup) in an average of 8 ± 1.5 s (mean ± SD; *N* = 574 flower visits), we considered that a flower’s reward was entirely “consumed” when a bee fed for more than 8 s before being joined on a flower. Accordingly, rewards were “not-consumed” (or partially consumed) when the bee fed on the flower for less than 8 s before being joined. As such, when joined by a nestmate a resident bee could choose to stay or leave from either an empty or partially emptied flower (Fig. 3).

To disentangle the effect of the time spent on a flower before an interaction and the duration of the interaction on the probability of a resident bee leaving a flower, we analyzed data in which direct contacts between bees occurred. We modelled the outcomes of interactions for the joining bee as a binomial distribution using a generalized linear mixed effect model (GLMM) with the *glmer* function in the R package ‘lme4’ [29], using the identities of the two bees as crossed random effects to account for repeated encounters between bees. Predictors used in interaction terms were standardized (mean centred and divided by their standard deviation) to allow main effects interpretation. We also added the difference in activity, experience and body size between the two interacting bees and their absolute values as covariates.

#### Effect of interactions on future foraging decisions

To quantify the impact of interactions on flowers on future flower visits, we modelled the number of visits made by each bee to each flower as a function of the interaction outcomes experienced by these bees on the same flower during the previous foraging bout. We applied GLMMs with Poisson distribution errors using the number of visits to flowers as a response variable, the binary outcome of interactions (leaving or staying on the flower) and the flower status (emptied or not-emptied) as fixed effects. We used the identities of the two bees as crossed random effects in the model.

## Results

### Bee-flower networks were highly modular

**Fig. 4:**
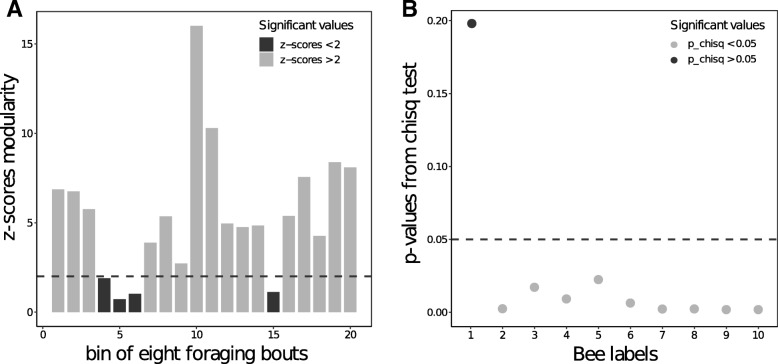
Network modularity and bee-flower persistence. **a** Modularity for observed bee-flower networks built on bins of eight foraging bouts compared to modularity calculated from random networks (null model; *N* = 20 networks). Modularity values for which z-scores are greater than two (grey columns) are significantly different from chance (black columns below the dashed horizontal line). **b** Persistence of bee-flower associations. Each data point represents the *p*-values obtained from the chi-square test of the number of times each bee is associated with each flower in their respective bee-flower module across the twenty bins of eight foraging bouts. Average specificity value for bees foraging during the same bin of eight foraging bouts. Bees using significantly persistent flowers across time (grey dots) have *p*-values lower than 0.05 (dotted line)

We identified 10 regular foragers in the colony (bees that made more than 20 flower visits within the five days of monitoring), that each made between 5 and 161 foraging bouts (foraging trips starting and ending at the colony nest box) and between 250 and 1279 visits to flowers. To study the dynamics of resource partitioning among these bees, we computed 20 bipartite networks (one network per bin of eight consecutive foraging bouts) and analysed variations of network modularity across these networks (Additional file 1: Figure S3). Modularity was higher than expected by chance (randomized networks) in 15 out of the 20 experimentally obtained networks (Fig. 4a). Bee-flower networks were therefore characterized by significant levels of resource partitioning among foragers. Network modularity was not affected by the number of bees foraging in a given time interval (Pearson’s correlation: *ρ* = − 0.27, df = 18, *p* = 0.24), which remained relatively constant across the study (mean ± SD: 7.95 bees ±1.43, *N* = 20 networks).

However, modularity was positively affected by the average foraging experience of bees (i.e. the cumulative number of flowers visited by a bee since the start of the experiment), so that the degree of flower overlap between bees decreased as foragers accumulated experience (LMM: *β* = − 0.001 ± 0.0004; t = 2.498; *p* = 0.025). We found no effect of foraging activity (i.e. sum of all flower visits within each bin of eight foraging bouts performed by a bee; LMM: *β* = − 0.004 ± 0.005; t = 0.833; *p* = 0.418) and the number of individuals foraging within the same interval (LMM: *β* = − 0.005 ± 0.004; t = − 1; *p* = 0.297).

### Flower specificity decreased with foraging activity and experience

**Fig. 5:**
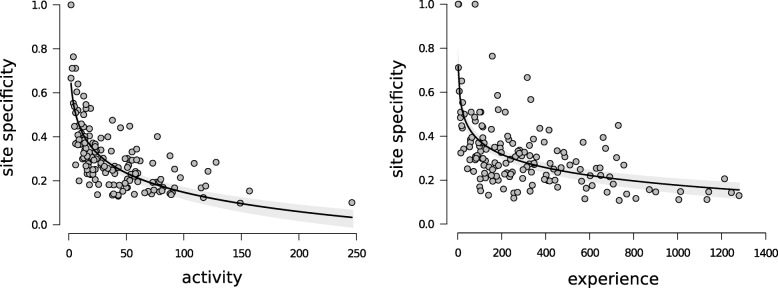
Flower specificity. Effects of **a** activity and **b** experience on flower specificity for each forager in each of the 20 bee-flower networks (*N* = 151). Black curves and grey shaded areas show the lines of best fit (logarithmic regression) and their standard errors respectively (see details in main text). Flower specificity values range from 0 (low flower specificity: a bee visits all 10 flowers) to 1 (high flower specificity: a bee visits only one flower). “Activity” is the sum of all flower visits within each bin of eight foraging bouts performed by a bee, and “Experience” is the cumulative activity of the bee during all its previous bins of eight foraging bouts (all flower visits since the start of the experiment)

The degree to which a bee used specific flowers (flower specificity) decreased with increasing foraging activity (LMM: *β* = − 0.111 ± 0.009; t = − 12.456; *p* < 0.001) and experience (LMM: *β* = − 0.032 ± 0.008; t = − 4.157; *p* < 0.001). Bees showed high inter-individual variability in flower specificity at low levels of activity and experience (i.e. during their first few foraging bouts), and low flower specificity at high levels of activity and experience (see Logarithmic relationship in Fig. 5a and b). While activity and experience were positively correlated (Pearson’s correlation: *ρ* = 0.35; t = 4.69; *p* < 0.001), these two measures had independent effects on flower specificity (i.e. co-linearity calculated using VIF = 1.68 for both predictors).

### Persistence of bee-flower associations

The associations between bees and flowers (i.e. network modules) were more stable than expected by chance. The difference between the observed and the expected frequencies of bee-flower associations across networks were significant for nine out of the ten bees (Fig. 4b; Additional file 1: Table S2), indicating that individual bees remained faithful to the same set of flowers across the five days of the study.

### Flower reward and time on a flower before interactions determined outcomes

Two bees interacted on flowers in 10% (*N* = 628) of all 6205 flower visits. Roughly half of these interactions (47.8%, *N* = 300) occurred on flowers containing no rewards. We identified four types of interactions dependent on whether the bee that landed second on the flower subsequently left either first (resident stays) or second (resident leaves), and whether the nectar reward of the flower was consumed or not. Mean and maximum duration of interactions on flowers were similar across the four types of interactions (Fig. 3).

Interestingly, 70% of these interactions (*N* = 440) involved physical contacts between bees, and the proportion of direct contacts was lower when the resident bee was replaced on a flower still containing nectar (resident stays on not-emptied flower in 49% of physical contacts on *N* = 181 interactions, Fig. 3). The probability of replacing a bee on a flower was lower if the nectar reward was not yet consumed (GLMM: *β* = − 1.491 ± 0.285; z = − 5.228; *p* < 0.001). This suggests that, during an encounter, resident bees tended to stay on flowers when the cup was full and leave flowers when the cup was empty.

The time a resident bee spent on a flower before being joined was a good predictor of interaction outcomes. Spending more time before the interaction occurred on a non-empty flower tended to result in the resident bee staying in most cases (probability of being replaced: GLMM: *β* = − 1.277 ± 0.643; z = − 1.987; *p* = 0.047). Accordingly, the probability of being replaced (resident leaves) on a non-empty flower was lower if the interaction lasted longer (GLMM: *β* = − 1.005 ± 0.432; z = − 2.328; *p* = 0.020). This effect was even stronger when accounting for the interaction between the time spent on a flower before the arrival of a nestmate and the reward status of the flower (GLMM: *β* = − 6.390 ± 2.299; z = − 2.780; *p* = 0.005). The probability of replacing a nestmate on a flower was not affected by differences in activity (GLMM: *β* = − 0.244 ± 0.319; z = − 0.765; *p* = 0.444), foraging experience (GLMM: *β* = − 0.561 ± 0.331; z = − 1.694; *p* = 0.090), flower specificity (GLMM: *β* = − 0.299 ± 0.336; z = − 0.888; *p* = 0.374), or body size (GLMM: *β* = 0.273 ± 0.502; z = 0.544; *p* = 0.683) between the two interacting bees. This suggests that resident bees had enough time to assess the reward status of the flower, which may have reinforced their motivation to stay and repel joining bees when floral rewards were still present.

### Outcomes of floral interactions influenced future flower visits

The number of visits to a flower increased if a bee pushed away a nestmate on that particular flower during the preceding foraging bout if the flower contained some rewards (GLMM, *β* = 0.32 ± 0.11; z = 2.81; *p* = 0.008). Previous experience of an encounter on the flower (GLMM, *β* = − 0.06 ± 0.19; z = 0.30; *p* = 0.381) or of flower reward status (GLMM, *β* = − 0.14 ± 0.28; z = 0.48; *p* = 0.355) alone had no impact on the bee’s future visits to that flower, suggesting that bees prioritized visits to flowers from which they had recently evicted a nestmate and obtained rewards.

## Discussion

Previous studies on intraspecific resource partitioning in bees indicate that foragers dynamically adjust the size of their foraging area in response to competition pressure [12–16]. Here we explored the consequences of these individual responses at the population level, by recording all flower visits made by ten foragers of a bumblebee colony during five consecutive days and applying bipartite network analyses to quantify the dynamics of resource partitioning among them. Our analyses show that the tendency of bees to specialize on individual flowers, and the outcomes of previous interactions on flowers, are key factors determining patterns of resource use by foragers.

Bumblebee foragers cooperate to provision their colony with nectar and pollen but exploit flower resources individually, with no recruitment to specific feeding locations [35, 36]. Foragers are therefore expected to spread out in order to maximise food collection rates at the individual and colony levels [12, 13]. Accordingly, in our experimental system, with a limited number of flowers promoting competitive interactions, we found significant levels of resource partitioning among bees associated with strong inter-individual variability in flower use. Some bees were more faithful to a subset of flowers (i.e. were more flower-specific), whereas others visited all flowers equally. This behavioural variability declined as bees became more active and gained foraging experience, a general pattern that may be explained by the fact that most bees started foraging on the same day (day 1 of the experiment) and gained experience with the experimental environment, flower design and flower spatial distribution synchronously.

Video data confirmed previous observations that bees occasionally interact on flowers [12], and suggest that these interactions may further favour resource partitioning. The first bee to arrive on a rewarding flower tended to keep the approaching nestmate away, a tendency that became even more pronounced when the time spent on the flower before the interaction was longer. Presumably, the resident bee had greater opportunity to assess the floral reward status, which increased its motivation to keep that flower for itself. This hypothesis is supported by the fact that the duration of interactions, between the resident and joining bee(s), lasted longer when flower reward was not yet consumed, suggesting bee firmness of resolve to hold that particular flower.

Importantly, these interactions on flowers seem to affect resource partitioning directly. Bees did not visit rewarding flowers more often than non-rewarding flowers during two consecutive foraging bouts, suggesting that in variable environments (such as our experimental array of flowers) learning flower reward values may not be a reliable strategy. However, after being kept away from a rewarding flower, bees decreased their number of visits to that particular flower during the subsequent foraging bout. Negative outcomes of interactions on flowers (i.e. being kept away from a profitable flower) might affect a bee’s motivation to return to the same flower, for instance through aversive learning [37].

Generally, the absence of any direct link between individual experience and the outcome of interactions seems to contrast with our previous observations on pairs of bumblebees showing that experienced foragers tended to evict newcomers from flowers, possibly to discourage them from exploiting resources in their established foraging area [12]. This difference may be explained because our new experiment was conducted with more bees over longer time scale, and all bees had the opportunity to begin exploring the flower array simultaneously (in contrast to [12] in which substantial differences in foraging experience were deliberately introduced by the experimental protocol).

Our results in a flight cage with high levels of competition suggest that resource partitioning can emerge from two main mechanisms: firstly, less experienced bees tend to be less active and remain more specific in their patterns of visitation to some flowers, while experienced bees tended to explore the environment more widely. Secondly, after negative outcomes from an interaction on a flower, bees show a lower tendency to return to that particular flower in future foraging bouts. Following these simple rules, bees might dynamically adjust their degree of resource partitioning depending on resource availability, potentially optimizing food collection rates at both the individual and colony level.

Although our study provides unprecedented levels of detail about bee foraging choices and interactions, it should be noted that these bees were observed in artificial conditions of high competition. Further studies are therefore needed to identify factors affecting resource partitioning by pollinators under more natural conditions. First, longer monitoring of bee movement patterns would inform whether these interactions equilibrate with regular turnover of foragers, as individuals from the colony get lost or die. From our results it can be expected that high levels of resource partitioning could naturally emerge, and be maintained, from variation in experience between naïve foragers that tend to visit a few flowers and more experienced foragers that tend to visit more flowers. Second, pollinators use reward level characteristics to make foraging decisions [20] and, if not constrained in an enclosed environment, bees might leave and find new unexploited flowers [22]. Third, in contrast to our artificial flowers, many natural flowers visited by bees are aggregated within inflorescences with many available nectaries [38] and lower nectar replenishment rates [39]. These factors may greatly reduce the frequency of physical interactions between foraging bees. Finally, whether similar competitive interactions would be observed between bees from different colonies, that do not share benefits of resource partitioning, is an open question. Newly developed tracking technologies to record the flight paths of bees in the field [6, 9, 12, 40], combined with further developments of network statistics, for instance using multi-layered network analyses to examine the influence of social interactions on spatial patterns [41], hold considerable promise to address these questions and shed light on the mechanisms underpinning space use by pollinators.

## Conclusions

We explored how all foragers from a bumblebee colony exploited an array of artificial flowers in a large flight cage using individual movement data and network analyses. Our results show that bees tended to minimize resource overlap as a consequence of individual experience and social interactions. This study in an artificial system suggests that efficient resource partitioning may emerge in natural conditions, in which bees can minimize competition and a natural turnover of foragers occurs.

## Additional files

Additional file 1: **Table S1.** Mean number of flowers visited per bee during each bin of eight foraging bouts, their standard deviations, as well as the maximum and minimum number of flowers. **Table S2.** Frequency of bee-flower associations obtained from network modules calculated using the DIRTLPAwb+ algorithm [31]. Rows refer to bee identity. Columns refer to flower identity. **Figure S1.** Experimental set-up. The outdoor flight cage (length = 44 m, width = 20 m, height = 3 m, mesh size = 0.5 mm) was erected on a flat pasture at the Centre for Agricultural Bioscience International (CABI) in Egham (Surrey, UK). Artificial flowers (F1-F10) with their webcams were connected to five laptop computers, each protected by a golf umbrella. A sixth laptop was used to power a webcam at the colony nest entrance. Picture by ML. **Figure S2.** Artificial flowers. An electric syringe pump filled with sucrose solution is connected through a plastic tube to the feeding cup (capacity = 40 μL). Sucrose solution (40% *v*/v) is pushed into the feeding cup at a constant rate (3.3 μL/min). Bees have access to the sucrose solution through a hole in the middle of the horizontal platform. A webcam (video camera connected to a laptop computer running motion sensitive software) pointing at the flower records each movement occurring on the landing platform. Each time a bee enters the camera’s field of view a video clip is recorded (minimum duration = 5 s) giving information about the identity of the bee (tag number), its arrival and departure time, and any interaction with other foragers on the flower. Picture by ML. **Figure S3.** Flower visitation matrices and computed modules. Y axis: flower identity. X axis: bee identity. Black-white gradients in each cell represent the frequency of visits made by each bee to each flower (darker colours denote higher visitation frequency). Complete visitation sequences can be found in dataset S1. Red polygons are the modules obtained from the DIRTLPAwb+ algorithm. (DOCX 4514 kb)

Additional file 2: **Dataset S1.** Raw dataset. Flower visitation dataset containing all flower visits by each individual bee over the five days of observation. (CSV 59 kb)

Additional file 3: **Video S1.** Example of “resident stays”. Bee Y67 landed on a flower occupied by bee W58 and left spontaneously. In this example, the flower was “not-emptied” as bee W58 fed on the flower for less than 8 s before the interaction occurred. (AVI 461 kb)

Additional file 4: **Video S2.** Example of “resident leaves”. Bee W25 landed on a flower occupied by bee Y35 and left second, therefore replacing bee Y35. In this example, the flower was “not-emptied” as bee Y35 fed on the flower for less than 8 s before the interaction occurred. (AVI 2069 kb)

## Abbreviations

df: Degree of freedom
GLMM: Generalized linear mixed effect model
GMT: Greenwich mean time
LMM: Linear mixed effect model
m: Metres
p: *p*-values
t: Student t-test statistic
z: Z-score statistic
*β*: Model estimate
μL: Microlitres
*ρ*: Pearson’s correlation coefficient
