# Supplementary material for: A spatial network analysis of resource partitioning between bumblebees foraging on artificial flowers in a flight cage
Source: Mov Ecol. 2019 Feb 21;7:4. doi: 10.1186/s40462-019-0150-z (PMC6383269; doi:10.1186/s40462-019-0150-z)
Supplement: Supplementary file 1 — Table S1. Mean number of flowers visited per bee during each bin of eight foraging bouts, their standard deviations, as well as the maximum and minimum number of flowers. Table S2. Frequency of bee-flower associations obtained from network modules calculated using the DIRTLPAwb+ algorithm [31]. Rows refer to bee identity. Columns refer to flower identity. Figure S1. Experimental set-up. The outdoor flight cage (length = 44 m, width = 20 m, height = 3 m, mesh size = 0.5 mm) was erected on a flat pasture at the Centre for Agricultural Bioscience International (CABI) in Egham (Surrey, UK). Artificial flowers (F1-F10) with their webcams were connected to five laptop computers, each protected by a golf umbrella. A sixth laptop was used to power a webcam at the colony nest entrance. Picture by ML. Figure S2. Artificial flowers. An electric syringe pump filled with sucrose solution is connected through a plastic tube to the feeding cup (capacity = 40 μL). Sucrose solution (40% v/v) is pushed into the feeding cup at a constant rate (3.3 μL/min). Bees have access to the sucrose solution through a hole in the middle of the horizontal platform. A webcam (video camera connected to a laptop computer running motion sensitive software) pointing at the flower records each movement occurring on the landing platform. Each time a bee enters the camera’s field of view a video clip is recorded (minimum duration = 5 s) giving information about the identity of the bee (tag number), its arrival and departure time, and any interaction with other foragers on the flower. Picture by ML. Figure S3. Flower visitation matrices and computed modules. Y axis: flower identity. X axis: bee identity. Black-white gradients in each cell represent the frequency of visits made by each bee to each flower (darker colours denote higher visitation frequency). Complete visitation sequences can be found in dataset S1. Red polygons are the modules obtained from the DIRTLPAwb+ algorithm. (DOCX 4514 kb) [file 40462_2019_150_MOESM1_ESM.docx]

**Supplementary information**

**A spatial network analysis of resource partitioning between bumblebees foraging on artificial flowers in a flight cage**

Cristian Pasquaretta^1^*, Raphael Jeanson^1^, Jerome Pansanel^2^, Nigel E. Raine^3,4^, Lars Chittka^5,6^, Mathieu Lihoreau^1^

^1^ Research Center on Animal Cognition (CRCA), Center for Integrative Biology (CBI); CNRS, University Paul Sabatier, Toulouse, France

^2^ Institut Pluridisciplinaire Hubert Curien, CNRS, Strasbourg, France

^3^ School of Biological Sciences, Royal Holloway University of London, Egham, TW20 0EX, UK

^4^ School of Environmental Sciences, University of Guelph, Guelph, Ontario, N1G 2W1, Canada

^5^ Department of Biological and Experimental Psychology, School of Biological and Chemical Sciences, Queen Mary University of London, Mile End Road, London E1 4NS, UK

^6^ Wissenschaftskolleg, Institute for Advanced Study, Wallotstrasse 19, 14193 Berlin, Germany

* Corresponding author - email: c[ristian.pasquaretta@univ-tlse3.fr](mailto:cristian.pasquaretta@univ-tlse3.fr)

**Dataset S1:** Raw dataset. Flower visitation dataset containing all flower visits by each individual bee over the five days of observation.

**Table S1:** Mean number of flowers visited per bee during each bin of eight foraging bouts, their standard deviations, as well as the maximum and minimum number of flowers.

|  | Number of flowers visited | |
| --- | --- | --- |
| Bin (of eight foraging bouts) | mean ± 1 SD | Range (min-max) |
| 1 | 4.87 ± 2.64 | 2-9 |
| 2 | 8.86 ± 1.68 | 6-10 |
| 3 | 6.20 ± 3.42 | 2-9 |
| 4 | 7.16 ± 1.94 | 5-10 |
| 5 | 6.33 ± 3.08 | 2-10 |
| 6 | 5.62 ± 3.58 | 1-10 |
| 7 | 7.80 ± 1.92 | 5-10 |
| 8 | 5.50 ± 2.07 | 3-8 |
| 9 | 8.00 ± 2.12 | 3-10 |
| 10 | 8.60 ± 1.35 | 6-10 |
| 11 | 7.90 ± 2.51 | 2-10 |
| 12 | 7.87 ± 1.64 | 6-10 |
| 13 | 6.43 ± 3.10 | 1-10 |
| 14 | 7.62 ± 1.51 | 6-10 |
| 15 | 7.33 ± 2.06 | 4-10 |
| 16 | 6.87 ± 2.42 | 4-10 |
| 17 | 8.00 ± 1.49 | 5-10 |
| 18 | 6.50 ± 2.88 | 2-10 |
| 19 | 8.25 ± 1.49 | 6-10 |
| 20 | 7.5 ± 2.20 | 5-10 |

**Table S2**: Frequency of bee-flower associations obtained from network modules calculated using the DIRTLPAwb+ algorithm [32]. Rows refer to bee identity. Columns refer to flower identity.

|  | **Flower identity** | | | | | | | | | |
| --- | --- | --- | --- | --- | --- | --- | --- | --- | --- | --- |
| **Bee identity** | **1** | **2** | **3** | **4** | **5** | **6** | **7** | **8** | **9** | **10** |
| **Bee 1** | 6 | 5 | 7 | 2 | 3 | 9 | 5 | 1 | 8 | 8 |
| **Bee 2** | 4 | 7 | 0 | 10 | 8 | 0 | 9 | 9 | 2 | 1 |
| **Bee 3** | 7 | 11 | 4 | 5 | 7 | 2 | 3 | 7 | 0 | 2 |
| **Bee 4** | 5 | 1 | 5 | 2 | 2 | 10 | 2 | 4 | 8 | 9 |
| **Bee 5** | 7 | 10 | 7 | 4 | 7 | 5 | 2 | 2 | 2 | 2 |
| **Bee 6** | 4 | 7 | 1 | 9 | 9 | 0 | 6 | 8 | 1 | 2 |
| **Bee 7** | 5 | 4 | 1 | 9 | 9 | 0 | 10 | 5 | 2 | 1 |
| **Bee 8** | 7 | 0 | 14 | 3 | 1 | 9 | 3 | 2 | 7 | 6 |
| **Bee 9** | 2 | 0 | 1 | 1 | 1 | 7 | 3 | 6 | 11 | 12 |
| **Bee 10** | 1 | 3 | 0 | 10 | 9 | 0 | 10 | 6 | 0 | 1 |

**Figure S1**


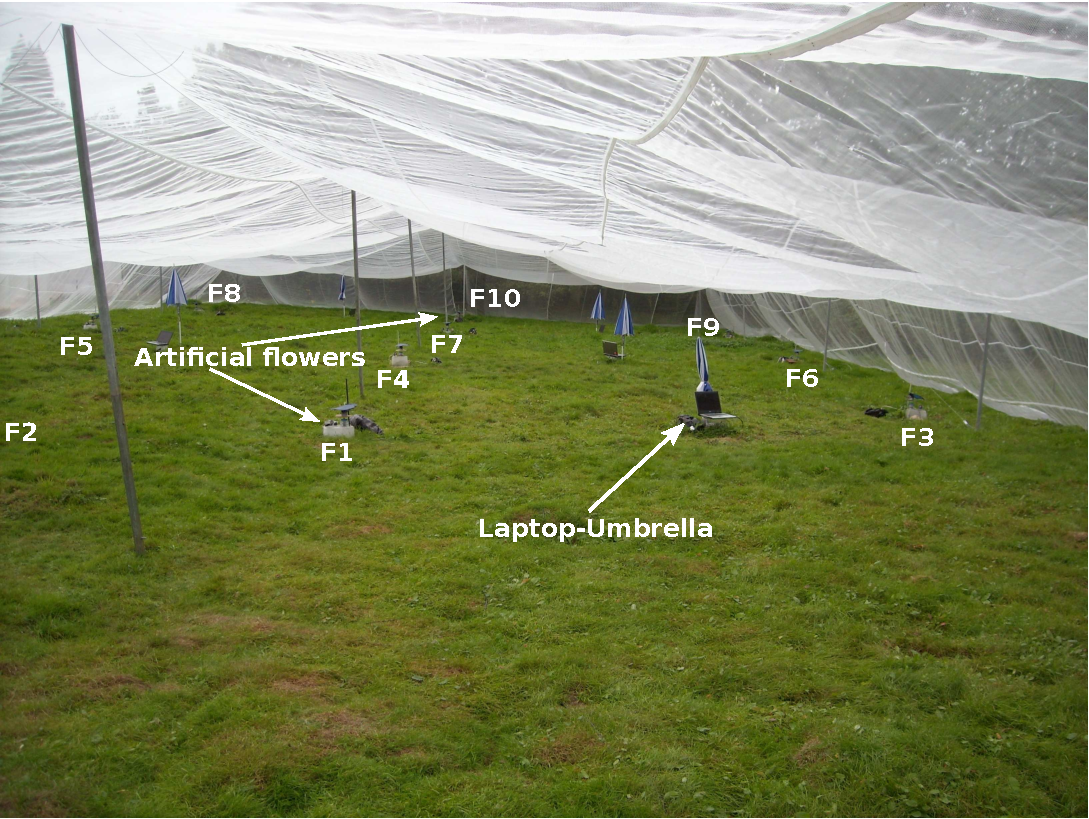


**Figure S1:** Experimental set-up. The outdoor flight cage (length = 44 m, width = 20 m, height = 3 m, mesh size = 0.5 mm) was erected on a flat pasture at the Centre for Agricultural Bioscience International (CABI) in Egham (Surrey, UK). Artificial flowers (F1-F10) with their webcams were connected to five laptop computers, each protected by a golf umbrella. A sixth laptop was used to power a webcam at the colony nest entrance. Picture by ML.

**Figure S2**


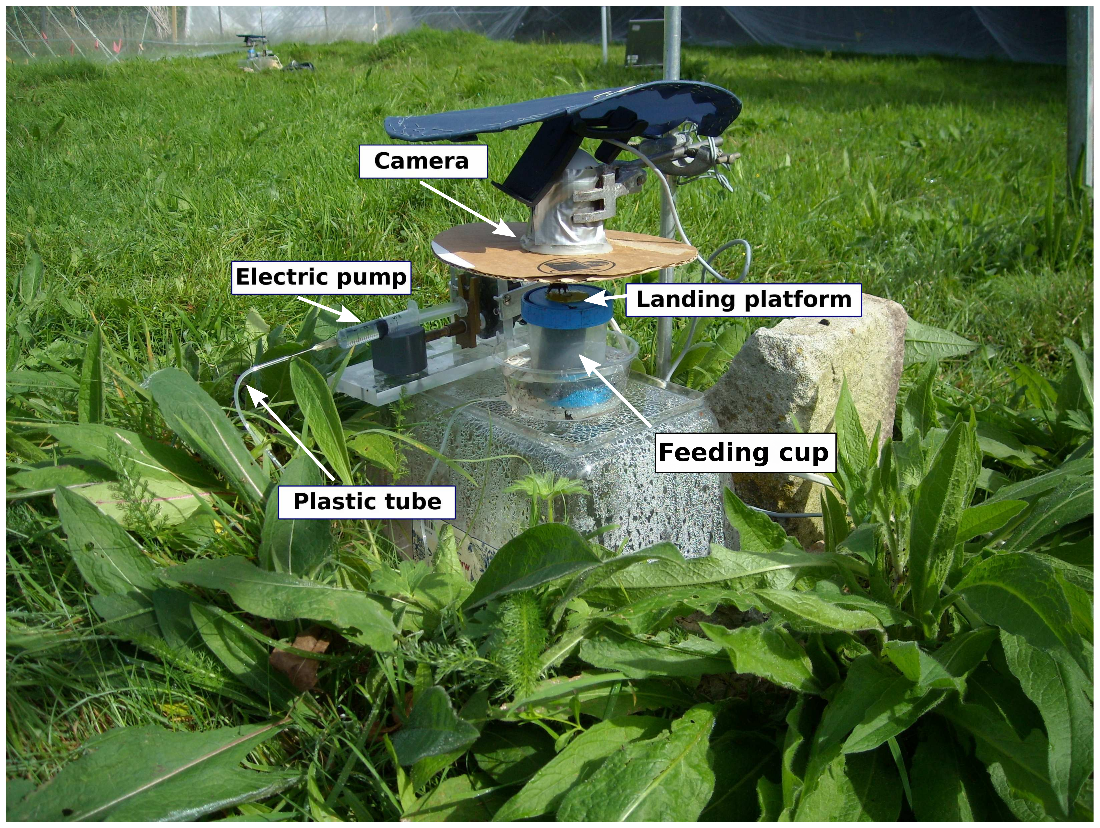


**Figure S2:** Artificial flowers. An electric syringe pump filled with sucrose solution is connected through a plastic tube to the feeding cup (capacity = 40 μL). Sucrose solution (40% v/v) is pushed into the feeding cup at a constant rate (3.3 μL/min). Bees have access to the sucrose solution through a hole in the middle of the horizontal platform. A webcam (video camera connected to a laptop computer running motion sensitive software) pointing at the flower records each movement occurring on the landing platform. Each time a bee enters the camera’s field of view a video clip is registered recorded (minimum duration = 5 s) giving information about the identity of the bee (tag number), its arrival and departure time, and any interaction with other foragers on the flower. Picture by ML.

**Figure S3**

**Figure S3:** Flower visitation matrices and computed modules. Y axis: flower identity. X axis: bee identity. Black-white gradients in each cell represent the frequency of visits made by each bee to each flower (darker colours denote higher visitation frequency). Complete visitation sequences can be found in dataset S1. Red polygons are the modules obtained from the DIRTLPAwb+ algorithm.

**Video S1:** Example of “join and leave”. Bee Y67 landed on a flower occupied by bee W58 and left spontaneously. In this example, the flower was “not-emptied” as bee W58 fed on the flower for less than 8 s before the interaction occurred.

**Video S2:** Example of “join and replace”. Bee W25 landed on a flower occupied by bee Y35 and left second, therefore replacing bee Y35. In this example, the flower was “not-emptied” as bee Y35 fed on the flower for less than 8 s before the interaction occurred.
